# Regional Variation of Gap Junctional Connections in the Mammalian Inner Retina

**DOI:** 10.3390/cells10092396

**Published:** 2021-09-12

**Authors:** Katalin Fusz, Tamás Kovács-Öller, Péter Kóbor, Edina Szabó-Meleg, Béla Völgyi, Péter Buzás, Ildikó Telkes

**Affiliations:** 1 Institute of Physiology, Medical School, University of Pécs, 7624 Pécs, Hungary; katalin.fusz@aok.pte.hu (K.F.); peter.kobor@aok.pte.hu (P.K.); ildiko.telkes@aok.pte.hu (I.T.); 2 Szentágothai Research Centre, University of Pécs, 7624 Pécs, Hungary; kovacs-oller.tamas@pte.hu (T.K.-Ö.); edina.meleg@aok.pte.hu (E.S.-M.); volgyi01@gamma.ttk.pte.hu (B.V.); 3 Centre for Neuroscience, University of Pécs, 7624 Pécs, Hungary; 4 MTA-PTE NAP-2 Retinal Electrical Synapses Research Group, 7624 Pécs, Hungary; 5 Institute of Biophysics, Medical School, University of Pécs, 7624 Pécs, Hungary; 6 Department of Experimental Zoology and Neurobiology, University of Pécs, 7624 Pécs, Hungary

**Keywords:** AII amacrine cell, Prox1, parvalbumin, gap junction, eccentricity, ON/OFF asymmetry

## Abstract

The retinas of many species show regional specialisations that are evident in the differences in the processing of visual input from different parts of the visual field. Regional specialisation is thought to reflect an adaptation to the natural visual environment, optical constraints, and lifestyle of the species. Yet, little is known about regional differences in synaptic circuitry. Here, we were interested in the topographical distribution of connexin-36 (Cx36), the major constituent of electrical synapses in the retina. We compared the retinas of mice, rats, and cats to include species with different patterns of regional specialisations in the analysis. First, we used the density of Prox1-immunoreactive amacrine cells as a marker of any regional specialisation, with higher cell density signifying more central regions. Double-labelling experiments showed that Prox1 is expressed in AII amacrine cells in all three species. Interestingly, large Cx36 plaques were attached to about 8–10% of Prox1-positive amacrine cell somata, suggesting the strong electrical coupling of pairs or small clusters of cell bodies. When analysing the regional changes in the volumetric density of Cx36-immunoreactive plaques, we found a tight correlation with the density of Prox1-expressing amacrine cells in the ON, but not in the OFF sublamina in all three species. The results suggest that the relative contribution of electrical synapses to the ON- and OFF-pathways of the retina changes with retinal location, which may contribute to functional ON/OFF asymmetries across the visual field.

## 1. Introduction

The retinas of most species show some type of regional specialisation that is evident in the distribution of photoreceptors and other cell types. Regional specialisations are thought to reflect an adaptation to the natural visual environment, optical constraints, and lifestyle of the species [1,2]. The best-known example is the peaking density of photoreceptors around the optical centre, such as in the fovea of primates [3] or area centralis of carnivores [4,5]. In the central regions, the neural circuitry is specialised for high-acuity vision, which in primates is also reflected by the number of cones converging on bipolar cells and eventually, on ganglion cells [6,7]. A number of perceptual functions also vary with visual field eccentricity, including not only visual acuity, but also contrast sensitivity, colour sensitivity, critical fusion frequency, motion perception, reaction time and crowding, many of which can be traced back to regional differences across the retina (see [8,9] for a review).

Electrical synapses contribute to diverse microcircuits in the retina that underlie a variety of functions, including the transmission of rod signals to ganglion cells [10,11,12,13,14], surround suppression [15,16], the synchronization of ganglion cell firing [17,18,19,20] and dynamic adaptation of these functions to changes in light level or circadian rhythms [21]. Connexin-36 (Cx36), the major constituent of mammalian retinal gap junctions, is positioned in key signal pathways [22]. For example, in the outer retina, gap junctions formed by Cx36 connect cone photoreceptors [13,23,24,25,26] with each other. Cx36 gap junctions connecting rods to cones form the secondary rod pathway [10,11], which is thought to be responsible for light detection at mid-scotopic intensity levels [14,27].

In the inner retina, gap junctions are found on all major cell types but interestingly, their involvement in ON and OFF pathways appears to be asymmetric. First, among the ON and OFF-sublaminae of the inner plexiform layer (IPL), the ON-sublamina contains more gap junctions [28]. There are two gap junction pathways that are formed exclusively in the ON sublamina, both involving AII amacrine cells. One of them is between AII cells and ON BCs and serves the signalling through the primary rod pathway. The second one connects AII cells into a dense homologous network [21,22,29,30,31,32,33]. There are four subpopulations of gap junctions maintained in the OFF sublamina, including two formed by retinal ganglion cells (RGCs) either with neighbouring RGCs or amacrine cells [34,35,36,37], a third population that connects amacrine cells to one another and a fourth population that exists between bipolar cells [38,39,40]. Three types out of these gap junctional subpopulations are abundant in the ON sublamina as well, including bipolar-bipolar [41], amacrine–amacrine [42,43,44,45,46] and RGC–amacrine [18,21,22,36] contacts. Direct RGC-RGC gap junctions, however, seem to be considerably less frequent here than in the OFF sublamina [36]. These microcircuits have been studied extensively at the local level, but their relationship to the topography of the retina is essentially unknown. Therefore, we were interested in obtaining a large-scale view of how the contribution of gap junctional connections to ON- and OFF-pathways changes across the retina.

Previously, we have observed a centre-periphery gradient in the density of Cx36 immunolabelled puncta in the ON-sublamina of the inner plexiform layer of cats [47]. In the present study, we extend these observations to any eccentricity-driven variation of inner retinal gap junction distribution in cats, rats and mice that represent species with different patterns of retinal specialisations. In addition, we compared the density of electrical contacts between the ON and OFF sublayers of the inner plexiform layer of all three animal models. In order to do this, plaque distributions were correlated with the AII amacrine cell coverage, as AII cells contribute considerably to gap junctional contacts in the ON, but not in the OFF sublaminae.

## 2. Materials and Methods

### 2.1. Animals and Sample Preparation

Retinas of 4 adult cats (*Felis catus*, 1 male aged 0.85 year, 3 females, aged 2.75, 8 and 8 years, respectively), retinas of 4 adult rats (*Rattus norvegicus*, Wistar, male, body weight 300–400 g) and retinas of 5 mice (*Mus musculus*), were used (males, 1–12 months old). Wild-type mice were from strain C57BL/6J (*n* = 3). The genetically modified animals referred to as PV-tdT (parvalbumin-tdTomato, *n* = 2) were cross-bred from PV-Cre (JAX #017320) and tdTomato (JAX #007909) at the animal house of the Szentágothai Research Centre, University of Pécs, Hungary. The sources of animals are listed in Table 1. The animals were kept, and the experiments were performed in accordance with Hungarian and European legislation. All procedures were approved by the Directorate for Food Chain Safety and Animal Health of the Baranya County Government Office, Hungary.

Cats were overdosed with 5% isoflurane followed by lethal injection of T61 (embutramide 250 mg/kg, tetracaine HCl 6.25 mg/kg, mebezonium iodide 63 mg/kg, Intervet, Boxmeer, The Netherlands) following unrelated physiological experiments. One animal was perfused intracardially with 4% paraformaldehyde in PBS (0.1 M phosphate-buffered saline, pH 7.5), the others were enucleated immediately after anaesthetic overdose. The eyes were cut along the *ora serrata* and the vitreous body was removed. The posterior eyecups were postfixed overnight at +4 °C and transferred into cold PBS. The retinas were prepared and cut into upper, lower, nasal, and temporal quadrants using the optic disk as the centre.

Rats were overdosed with 5% isoflurane and perfused intracardially with 4% paraformaldehyde in PBS. The eyes were cut along the *ora serrata* and the lens and vitreous body were removed. The posterior eyecups were postfixed overnight at +4 °C in 4% paraformaldehyde in PBS and the retinas were prepared in cold PBS.

Mice were sacrificed after isoflurane anaesthesia (0.2 mL/l) by cervical dislocation. The eyes were removed immediately after termination. Eyeballs were cut at the *ora serrata*, then the lens and vitreous body were removed. Retinas were fixed in 4% paraformaldehyde in PBS at room temperature for 15 min.

### 2.2. Immunohistochemistry and Confocal Microscopy

Free-floating retinal quadrants or whole retinas were first incubated with a blocking solution composed of 10% normal goat serum in antibody diluting solution (0.25% bovine serum albumin, 0.001% sodium azide, and 0.2% Triton X-100 in 0.1 M PBS) for 2 days. The same solution was used for all further antibodies unless stated otherwise. Tissue samples were then incubated with the primary antibodies at +4 °C for 4 days using the following dilutions: polyclonal anti-calretinin (CaR) produced in rabbit, 1:2000; monoclonal anti-Cx36 produced in mouse, 1:1000; polyclonal anti-Prox1 produced in rabbit, 1:250; monoclonal anti-parvalbumin produced in mouse, 1:600; monoclonal anti-calbindin produced in mouse, 1:100.

The following steps were done at +4 °C overnight. Cx36 immunoreactivity was visualized using biotinylated anti-mouse IgG (H + L) (1:100 dilution) followed by streptavidin-Alexa Fluor 488 conjugate (1:200 dilution in 0.1 M PBS). CaR immunoreactivity was visualised with goat anti-rabbit antibody Texas Red conjugate (1:100 dilution). Prox1 immunoreactivity was visualised with donkey anti-rabbit Alexa Fluor 594 (1:500 dilution), parvalbumin (PV) and calbindin (CaB) immunoreactivity were visualized with donkey anti-mouse Alexa Fluor 488 (1:500 dilution). The sources of antibodies are listed in Table 1.

We washed the retinal pieces between the incubations five times for 10 min in PBS. Retinal pieces were mounted in Aqua-PolyMount (rats, cats; Polysciences, Warrington, PA, USA) or VectaShield (mice; Vector Labs., Burlingame, California, USA) media with the ganglion cell layer facing the coverslip.

We inspected the flat-mounted retinas using a Zeiss LSM 710 confocal laser scanning microscope (Carl Zeiss, Jena, Germany) through a Plan-Apochromat 63× objective lens (NA 1.4), following a tile-scan at 10× for localisation. We took confocal stacks at selected regions of interest (ROIs, see below); the horizontal size of the ROIs was 135 × 135 μm and the z-stacks spanned depth from the outer nuclear layer to the optic fibres (for Cx36 density measurements) or a narrower range as needed for inspection of histological structures. Voxel size was 0.132 μm × 0.132 μm × 0.381 μm or 0.132 μm × 0.132 μm × 0.5 μm.

### 2.3. Measurement of Retinal Eccentricity and Feature Density

Regions of interest (ROIs) were selected randomly to cover eccentricities as equally as possible. To calculate eccentricity in cat retinas, first the location of each ROI was measured in polar coordinates with the optic disk as the centre (range of distances from the optic disk 0.16–14.2 mm; mean ± SD 8.14 ± 3.74 mm). These coordinates were then converted into Cartesian coordinates of the entire flattened retina, taking advantage of the fact that neighbouring quadrants had common edges. The eccentricity of each ROI was calculated from the position 3 mm lateral from the optic disk; this position is in good agreement with the location of the area centralis in the cat retina [48,49]. The eccentricities of ROIs were statistically not different in the four quadrants (Kruskal–Wallis test, *p* > 0.05). For mouse retinas, we only determined eccentricity categories of the ROIs (centre, mid-centre, periphery, Supplementary Figure S1).

To determine cell densities, we used the Fiji distribution of the ImageJ software package [50]. Cell bodies of the cell type of interest were identified based on their immunohistochemical labelling and laminar position and each member of the cell type found in a z-stack was marked using the Cell Counter plugin. Their number was divided by the area of the ROI to obtain an areal density in mm^–2^.

Cx36 plaques were identified using the 3D View-Surfaces module of the Imaris image processing software (Oxford Instruments, Zurich, Switzerland). This module fitted irregular solid shapes to aggregates of voxels showing increased Cx36 immunolabel. We used the following settings: Thresholding method, Background Subtraction; Diameter of the largest sphere that fits into the object: 0.1 μm. Based on their 3D coordinates, we assigned each Cx36 plaque to one of the 5 strata of the IPL. The Z coordinate ranges of the strata were determined by dividing the Z-distance between the limits of the IPL into five equal parts. Strata 1–2 were assigned to the OFF- sublamina and strata 3–5 were assigned to the ON-sublamina. This also allowed us to calculate the volume of each sublamina for each ROI. We then calculated the volumetric density of the Cx36 plaques by dividing their number found in a ROI by the volume of the IPL sublamina in that ROI. Only Cx36 plaques with a volume between 0.02 µm^3^ and 2.5 µm^3^ were counted to reduce the effect of pixel noise and to exclude objects too large for a Cx36 plaque.

### 2.4. 3D Colocalization Analysis

To show parvalbumin-tdTomato (PV-tdT) and Prox1 colocalization, a selected area was scanned by the confocal microscope with a 63x-oil objective at high resolution (voxel size of 0.15 μm × 0.15 μm × 0.4 μm). A side-view rotation was then rendered from this Z-stack using Fiji’s ‘3D viewer’ module. Four smaller ROIs (20 μm × 20 μm) containing selected amacrine cells were cut (three with evident colocalization and one with no PV-tdT label only) for subsequential rotations to show dendritic morphology. Intensity profiles comparing Prox1, Cx36 and tdT label along line segments were also produced in Fiji.

### 2.5. Data Analysis

ANOVA, correlation analysis, linear regression and statistical tests were done in IBM SPSS (IBM Corporation, Armonk, NY, USA). Significance level was set to *p* < 0.05.

## 3. Results

### 3.1. Prox1 Immunoreactive Cell Types in Cat, Rat and Mouse Retinas

The homeodomain protein Prox1 is expressed in horizontal cells, bipolar cells and AII amacrine cells of the adult mouse retina [51,52]. Here, we begin with testing the hypothesis that Prox1 is present in the same cell populations of rats as well as cats.

**Figure 1:**
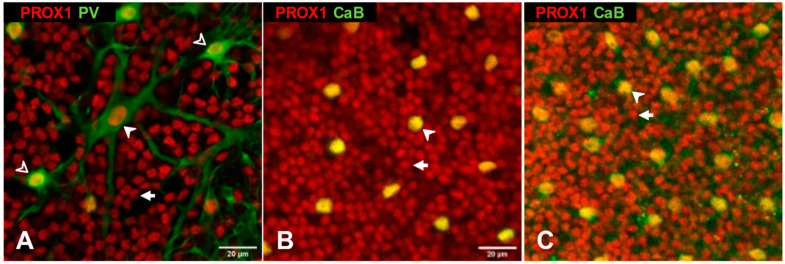
Prox1 immunoreactive cell nuclei (red channel) in the inner nuclear layer of cat (**A**), rat (**B**) and mouse (**C**) retina. Images show a horizontal view of whole-mount preparations at the level of horizontal cells. Nuclei of two types of cells are labelled in each species; larger and sparser horizontal cells (arrowheads) and numerous small bipolar cells (arrows). **A,** In the cat, Prox1 colocalizes with parvalbumin (green channel), which labels somata and dendrites of both A- (filled arrowhead) and B-type (open arrowheads) horizontal cells. In rat (**B**) and mouse (**C**) retina, Prox1 colocalizes with calbindin in the somata of horizontal cells. Scale bar 20 μm.

Figure 1 shows retinal whole mounts immunostained for Prox1 with the focal plane on the outer aspect of the inner nuclear layer (INL). Two types of cell nuclei expressing Prox1 could be readily distinguished. One population was relatively sparse (cell densities in cat: 485 ± 80 mm^−2^, *n* = 9 z-stacks; rat: 838 ± 314 mm^−2^, *n* = 15; mouse: 510 ± 102 mm^−2^, *n* = 20), had larger nuclei (horizontal diameters for cat: 9.65 ± 0.29 μm, *n* = 9; rat: 8.27 ± 0.14 μm, *n* = 15; mouse: 9.33 ± 0.19 μm, *n* = 21) and was more intensely stained. In cat retinas, these cells also contained parvalbumin (PV, Figure 1A), a marker of A- and B-type horizontal cells [53,54]. PV immunoreactivity also revealed the dendritic trees so that type-A (larger somata, stouter proximal dendrites) and type-B (smaller somata, thinner proximal dendrites) cells could be distinguished confirming that both horizontal cell types are Prox1 positive in the cat retina. In rat and mouse retinas, there is a single type of horizontal cell [55], which can be identified through calbindin (CaB) immunoreactivity [56] and by their laminar position. We found that all CaB-immunoreactive horizontal cells also contain Prox1 in both rodent species (Figure 1B,C).

Prox1 immunostaining also revealed numerous smaller (cat: 6.09 ± 0.09 μm, *n* = 238, *p* < 0.001, rat: 5.33 ± 0.14 μm, *n* = 344, *p* < 0.001, mouse: 6.30 ± 0.11 μm, *n* = 379, *p* < 0.001 in t-test against horizontal cell profiles) and somewhat less strongly labelled cell nuclei, which did not contain PV or CaB (Figure 1). Their laminar location, high density (cat: 9573 ± 1276 mm^−2^, *n* = 3 z-stacks; rat: 14,222 ± 1299 mm^−2^, *n* = 3; mouse: 9340 ± 335 mm^−2^, *n* = 3), and size were similar in all three species, suggesting that many bipolar cells are Prox1-positive in cats and rats just like in the mouse retina [52].

**Figure 2:**
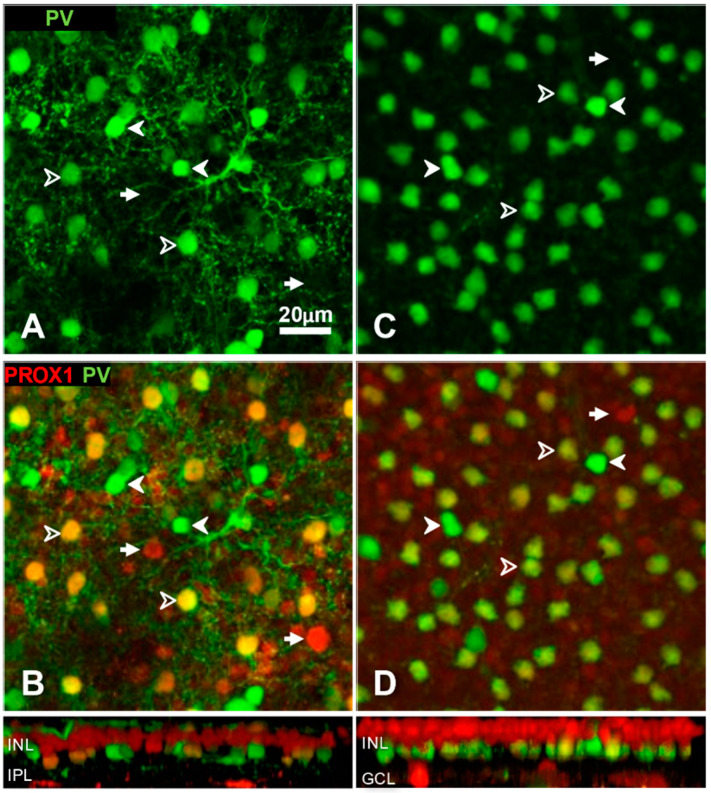
Typing of amacrine cells based on double labelling with parvalbumin (green channel) and Prox1 (red channel) immunoreactivity in cat (**A**,**B**) and rat (**C**,**D**) retina. Microscopic images show horizontal views of whole-mount preparations at the level of amacrine cells. Side-view reconstructions of representative sub-volumes spanning the INL (top) IPL and ganglion cell layer (GCL) are shown below (**B**,**D**). (**A**,**C**) Anti-parvalbumin antibody labelled two populations of amacrine cells in both species. The weakly parvalbumin-positive neurons (open arrowheads) include (in cats, A) or are exclusively (in rats C) AII amacrine cells. B, D. The combination of parvalbumin and Prox1 immunolabels reveals three amacrine cell types in both species. Amacrine cells with a strong PV expression are Prox1-negative. Weakly PV-positive amacrine cells are Prox-1 positive. A small population of Prox1-positive amacrine cells is PV-negative (arrows). Scale, 20 μm.

**Figure 3:**
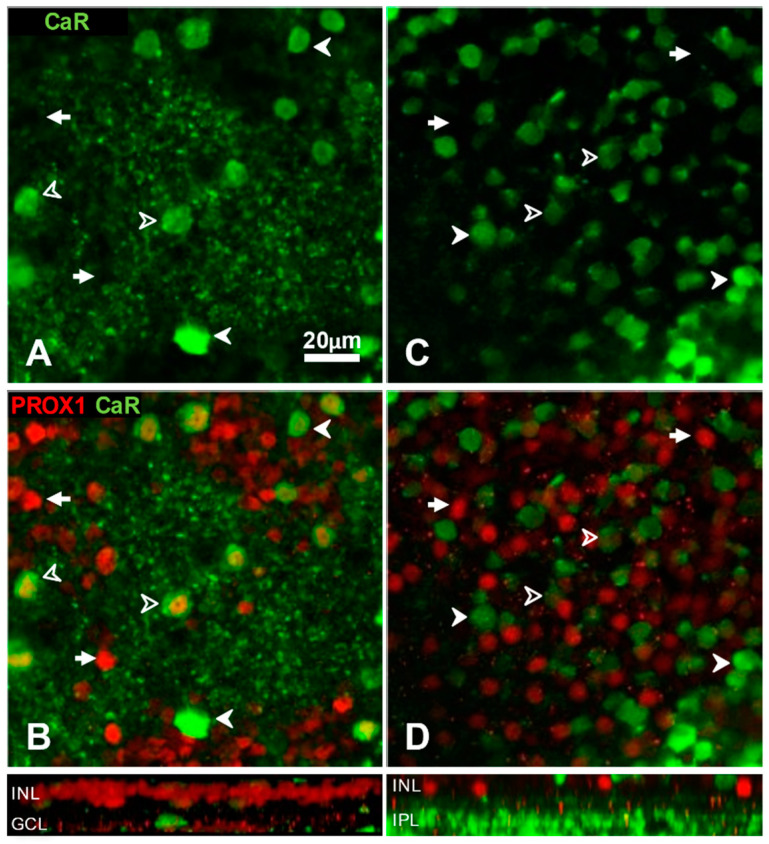
Typing of amacrine cells based on double labelling with calretinin (CaR, green channel) and Prox1 (red channel) immunoreactivity in cat (**A**,**B**) and rat (**C**,**D**) retina. Microscopic images show horizontal views of whole-mount preparations at the level of amacrine cells. Side-view reconstructions of representative sub-volumes spanning the INL (top) IPL and ganglion cell layer (GCL) are shown below B and D. In cat retina (**A**,**B**), the strongest CaR-immunoreactivity (**A**) is seen in sparsely distributed amacrine cells (solid arrowheads), which are Prox1-negative (**B**). AII amacrine cells correspond to the weakly CaR-immunoreactive cells (open arrowheads), which are more numerous and always Prox1 immunoreactive (**B**). Some amacrine cell bodies expressed only Prox1 and no CaR in the cat (arrows). The cell-free regions filled with PV-labelled varicosities belong to the IPL. In rat retina (**C**,**D**), the anti-CaR antibody did not differentiate amacrine cell types clearly (**C**). A combination with Prox1 immunolabel (**D**) revealed that the two markers label largely non-overlapping amacrine cell populations, with the majority showing either CaR (solid arrowheads) or Prox1 immunoreactivity (arrows) and only a few are double-labelled (open arrowheads).

The proximal INL also contained Prox1 immunopositive cells in each species. These cells appeared more heterogeneous in size and staining intensity than the horizontal cells or bipolar cells described above. According to previous reports on mouse retina, the majority or maybe all of the Prox1 positive cells in this layer are AII amacrine cells [52]. In cat and rat retinas, we used double labelling with an anti-PV (Figure 2) and anti-calretinin (CaR, Figure 3) antibodies to further identify Prox1 expressing amacrine cells.

In both species, PV labelling revealed two types of amacrine cells, which corresponded to earlier descriptions [54,57,58]. Strongly PV-immunoreactive amacrines were sparser (8.9% out of 325 labelled amacrine cells belonged to this group in cat retinas, and the same ratio was 6.2% out of 2544 cells in rat retinas). Their cell bodies emitted one or two main dendrites whose branches could be followed for at least 50 μm (Figure 2A); thus, they fall in the category of small-field or larger amacrine cells [59]. Typical members of this group did not express Prox1 (Figure 2B,D).

The second PV-immunopositive amacrine cell group was less intensely labelled and more numerous (Figure 2A,C; frequency 24.3% and 90.5% of all labelled cells in cat and rat retinas, respectively). In rats, these cells have been identified as AII amacrine cells [57]; in cat retina, the majority of them are also AII cells [54,58]. These amacrine cells also contained Prox1 immunoreactivity (Figure 2B,D).

Calretinin is known to be a marker of largely different amacrine cell types in cats and rats. In cats, most of the CaR-containing amacrines are of type AII along with a sparser, more strongly labelled population [28,47,56,58,60]. Our current experiments showed that CaR-positive AII amacrine cells are always Prox1-immunoreactive (Figure 3A,B). In rat retina, CaR is a marker of non-AII (mainly starburst) amacrine cells [61,62]. In our CaR and Prox1 double-labelled rat retinas, the two markers revealed in essence, complementary populations with only a few double labelled cells present (Figure 3C,D), which is compatible with our assumption that Prox1 labels AII amacrines in the rat.

In both PV- and CaR-labelled retinas, some of the amacrine cell bodies were revealed by the anti-Prox1 antibody alone (Figure 2B,D and Figure 3B,D). Without further neurochemical markers and any labelling in their dendrites, these cells could not be assigned to known subtypes. This population was more abundant in cat retinas (e.g., 66.8% of all labelled cells in the PV + Prox1 labelled material vs. 3.3% in rodent retinas), and included many that were more lightly labelled and seemed difficult to differentiate from Prox1-immunoreactive bipolar cells (Figure 3B and Figure 6A). Consequently, the density estimates of Prox1-positive amacrine cells were more uncertain and therefore were not used as a proxy for retinal location in our analysis of cat retinas (see below).

**Figure 4:**
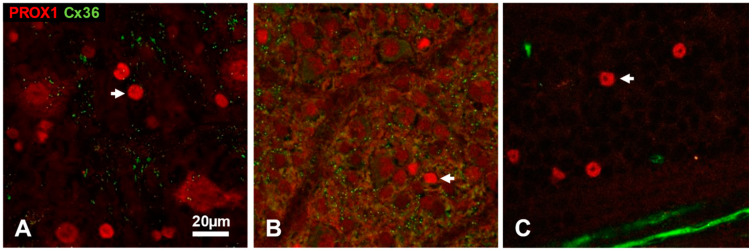
Prox1 immunoreactive cell nuclei (red channel) in the ganglion cell layer of flat mounted cat (**A**), rat (**B**) and mouse (**C**) retina. Smaller nuclei (arrows) are putative displaced amacrine cells (see details in the text). In the cat retina (**A**), some ganglion cell somata are also Prox1-immunoreactive. Connexin-36 puncta appear in irregular patches where the neuropil of the inner plexiform layer intrudes between cell bodies, but no somatic plaques can be observed on the Prox1-positive cells. Non-specific labelling by the anti-Cx36 antibody is seen in a bundle of optic fibers in the mouse retina (**C**). Scale bar 20 μm.

Whereas the majority of Prox1-expressing neurons were in the INL, some strongly Prox1-positive cell bodies were also found in the ganglion cell layer in cats as well as rodents (Figure 4). Their size was similar to the Prox1+ regular amacrine cells (cat: 7.37 ± 0.16 μm, *n* = 8, *p* = 0.13, rat: 8.86 ± 0.33 μm, *n* = 6, *p* = 0.08, mouse: 7.97 ± 0.22 μm, *n* = 6, *p* = 0.81 in t-test against Prox1-positive regular amacrine cells), suggesting they are displaced amacrine cells. Only in the cat did some ganglion cells show Prox1-immunoreactivity, which was rather weak and unusually appeared also in the cytoplasm (Figure 4A).

### 3.2. Identification of Individual AII Amacrine Cells in the PV-tdT Mouse Line

To further validate the Prox1 immunoreactive cell’s background in the INL, we used the PV-tdT mouse line to show the morphology of these cells. Although PV expression is known to be limited to the GCs (and an unidentified group of amacrine cells) in mice [63,64], in this mouse line, tdT red-fluorescent reporter is expressed sporadically in some isolated amacrine cells, probably due to embryonal expression of Cre-recombinase. Expression of the fluorescent tdT protein allowed us to observe the morphology of these amacrine cells in fine detail.

**Figure 5:**
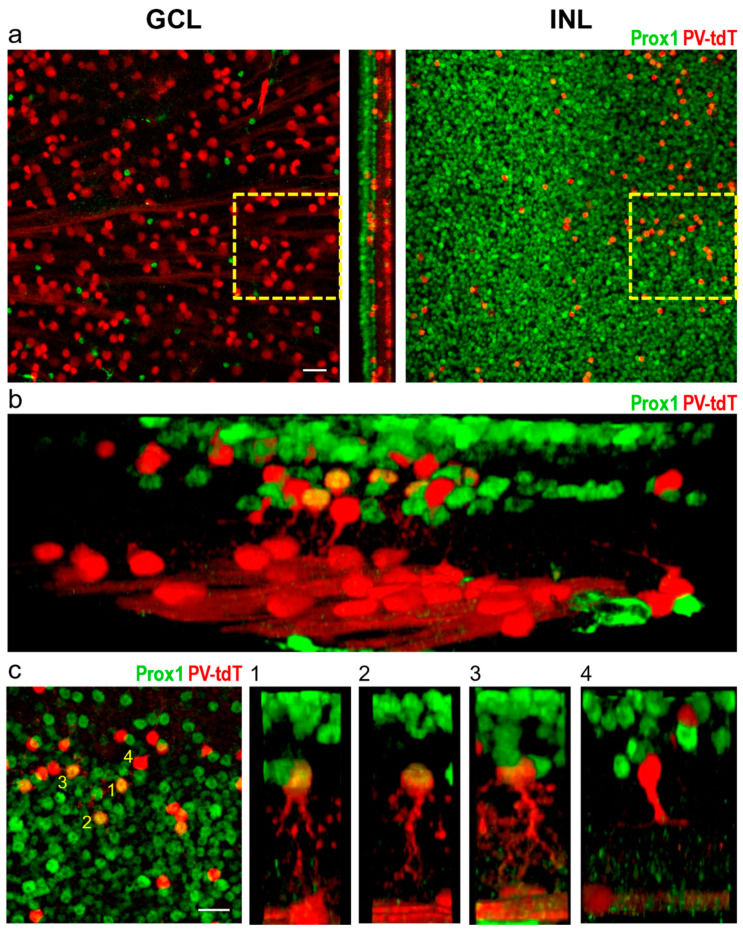
Colocalization of tdT and Prox1 in whole-mount preparation of the PV-tdT mouse retina. Overview images (**a**) show no colocalization of Prox1 with tdT in the ganglion cell layer (GCL), but in the INL, patches of amacrine cell bodies were double-labelled. The box outlined in yellow broken lines is shown at higher magnification in **c**. Three-dimensionally rendered and rotated views (**b**,**c**) of double labelled cells revealed their typical AII-like dendritic morphology (**c**(**1**–**3**)). Prox1-negative amacrine cells showed sparser, monostratified dendritic arbours reminiscent of wide-field amacrine cells (**c**(**4**)). Numbers in the left panel of **c** identify cells shown on side-views numbered 1 through 4, Scale = 25 μm.

Following immunolabelling of PV-tdT mouse retina with anti-Prox1 antibody (Figure 5a,b), we found that double-labelled neurons were exclusively amacrine cells, but only a small fraction of Prox1 immunoreactive amacrine cells (3.17% measured from six 224 × 224 μm regions on one retina)expressed tdT. They showed consistent morphology with axially ovoid somata, short, lobular dendrites in the OFF-sublamina and longer, thinner transversal dendrites projecting to the ON sublamina of the IPL, characteristics of AII cells (Figure 5b,c; [65,66,67]). Many of the tdT-labelled amacrine cells, however, were negative for Prox1 (29.67 cells per region on average). An example is shown in Figure 5c(4); this neuron had long, monostratified dendrites, suggesting that it was a wide-field amacrine cell. Taken together, these results corroborate earlier findings that Prox1 is a specific marker of AII cells [52].

### 3.3. Somatic Cx36 Plaques on Amacrine Cells of the Cat, Rat and Mouse Retina

**Figure 6:**
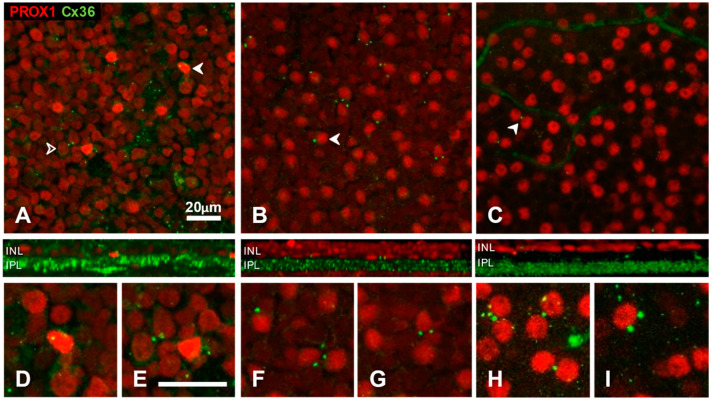
Somatic Cx36 plaques on Prox1 immunoreactive amacrine cells in whole-mount preparations of cat (**A**,**D**,**E**), rat (**B**,**F**,**G**) and mouse (**C**,**H**,**I**) retinas. Red channel, Prox1; green channel, Cx36. Side-view reconstructions of representative sub-volumes spanning the proximal INL (top) and IPL are shown below (**A**–**C**). Somatic Cx36 plaques were found in close apposition to a subset of Prox1-immunoreactive cell bodies. Examples are marked by arrowheads or shown at higher magnification in (**D**–**I**). In the cat retina (**A**), somatic plaques occurred in apposition to both strongly Prox1 positive (solid arrowheads) and lightly Prox1-positive (open arrowhead) cells.

In the following section, we turn our attention to the relationship of Prox1 immunoreactive cells to Cx36 immunoreactive punctate structures, the light-microscopic correlates of gap junctions. We begin our analysis with the inner nuclear layer. The INL is of interest because in previous studies, we saw large Cx36 plaques on cell bodies of some AII amacrine cells of cat [47] or mouse [68] retina. In our current material, we surveyed Prox1-positive amacrine cells systematically for the presence of Cx36 plaques (Figure 6). For this, we used optical sections focussed on the inner aspect of the INL and excluded regions where the neuropil of the IPL intruded between cell bodies.

Although Prox1 immunoreactivity is limited to the nuclei, and cell boundaries are not observable, plaques in the INL were almost exclusively in close apposition to Prox1-positive profiles, a few of the plaques sometimes surrounded the labelled nucleus (Figure 6D–I). The proportion of somatic Cx36 plaques and Prox1 immunoreactive amacrine cells was 8% in cat (*n* = 163 cells), 10% in rat (*n* = 103 cells) and 11% in mouse retina (*n* = 131 cells). In the cat retina (Figure 6A), somatic plaques were three times more frequent on strongly Prox1-immunoreactive amacrine cells (18% out of 470 cells) than they were on their weakly labelled counterparts (6% of cells). No somatic plaques could be observed on Prox1-positive neurons of the ganglion cell layer in either species (Figure 4).

The presence of such Cx36 plaques suggests a route for electrical coupling through the cell bodies of AII (and likely other Prox1 expressing) amacrine cells. The synaptic partners can be other amacrine cells of the same kind, since the somatic plaques were sometimes seen at the confluence of Prox1 positive cell bodies (Figure 6E–H). However, the neighbouring cell next to the plaque was quite often unstained (Figure 6A–C,I), raising the possibility of somatic coupling to other cell types.

**Figure 7:**
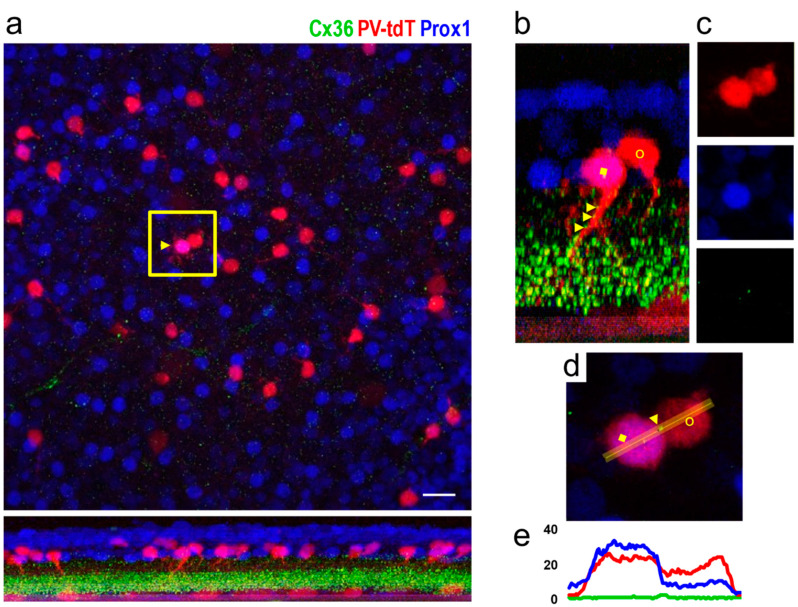
Colocalization of tdT, Prox1 and Cx36 immunofluorescence in amacrine cell somata of PV-tdT mouse retina shown in a tangential view at the level of the amacrine cell bodies (top of panel (**a**)) and as an orthogonal section along the line indicated by the yellow arrowhead (bottom of panel (**a**)). The region enclosed by the yellow box contains an AII amacrine cell (yellow arrowhead, magenta color due to double labelling with Prox1 and tdT) along with a tdT-positive neighbouring non-AII amacrine cell (red). The corresponding dendritic morphology of these cells is readily seen on the side-view in (**b**) (yellow square, AII amacrine cell; yellow circle, non-AII amacrine cell). Panel (**c**) shows the boxed region from **a** with the three channels separated (PV-tdT, Prox1 and Cx36 from top to bottom). Note the dot on the green channel, which indicates a Cx36 plaque where the two cell bodies touch each other. A close-up of this region (**d**) and an intensity profile, measured across the Cx36 plaque (**e**) confirm the close apposition of the plaque to both cell bodies. Some Cx36-puncta are also present on the proximal dendrite of the AII cell (**b**, yellow arrowheads). Markers as in (**b**). Scale, 25 μm.

We have addressed the question of heterocellular coupling in transgenic PV-tdT mouse retinas. Here, we identified Cx36 plaques between Prox1 positive (AII) and Prox1 negative (non-AII) amacrine cells that were revealed by tdT fluorescence (Figure 7). The detailed dendritic morphology revealed by tdT expression allowed us to confirm the existence of gap junctions between the cell bodies of different amacrine cell types.

### 3.4. Regional Variation of Connexin-36 Density in the OFF- and ON-Sublaminae of the Inner Plexiform Layer

In this part of the study, we sought to detect large scale variations in gap junction density with retinal location and compare this variation between the ON and OFF sublaminae of the IPL in cats, rats and mice. Regional specialisation is evident in the distribution of photoreceptors and several neuron types, but the topography of the cell distributions is different in cats and rodents (cat [4,69]; mouse [70]), it may have complicated shapes and also vary by individual [71,72,73]. Therefore, we decided to use cell density as a general measure of regional variation in rodents, instead of distance from some retinal landmark. In the following, high cell density has thus the same meaning as central location of a concentrically organised retina, and conversely, low cell density is equivalent to a peripheral location.

**Figure 8:**
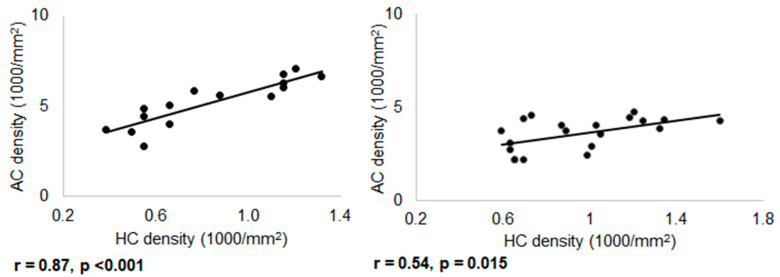
Correlation of density of Prox1 immunoreactive amacrine and horizontal cells in rat (*n* = 15) and mouse retina (*n* = 20). A strong positive correlation was found in both cases (r = 0.87, *p* < 0.001 and r = 0.54, *p* = 0.015).

The densities of many cell types might be used as a surrogate for retinal location. Regarding Prox1-immunoreactive amacrine cells and horizontal cells, we found that their densities change with location, and they are positively correlated (rat, r = 0.87, *p* < 0.001; mice, r = 0.54, *p* = 0.015; Figure 8). It is important to note that mean densities for Prox1-positive amacrine cells (5085 ± 1139 for rats, 3687 ± 904 and 3520 ± 836 for wild type and PV-tdT mice, Table 2) were very much in line with the reported densities of AII amacrine cells in these species [52,57]. This supports the notion that the population of Prox1-positive amacrine cells largely overlaps with AII cells.

In the following analysis, we used either Prox1-positive amacrine cell density, or in one rat retina, CaR-positive amacrine cell density as a measure of retinal location. As described above, CaR is the marker of a large population of non-AII amacrine cells in the rat retina that includes the cholinergic starburst cells [61,62]. The densities of starburst and AII amacrine cells do, however, follow similar regional distribution patterns to each other [57,74].

Our previous analysis showed that Cx36 plaque density correlates with the density of AII amacrine cells so that central regions show a higher density of both features compared with peripheral regions [47], but this analysis was limited to the ON IPL of the cat retina. The OFF sublamina contains generally fewer gap junctions in the mammalian retinas studied so far [28]. This was confirmed in our material where the OFF sublamina contained significantly fewer Cx36 plaques per mm^3^ than the ON sublamina (*p* < 0.05, Table 2). Here, we calculated plaque density per unit volume to eliminate the effect of the different thicknesses of the layers, which exist between the ON and OFF sublaminae, between species or retinal locations.

**Figure 9:**
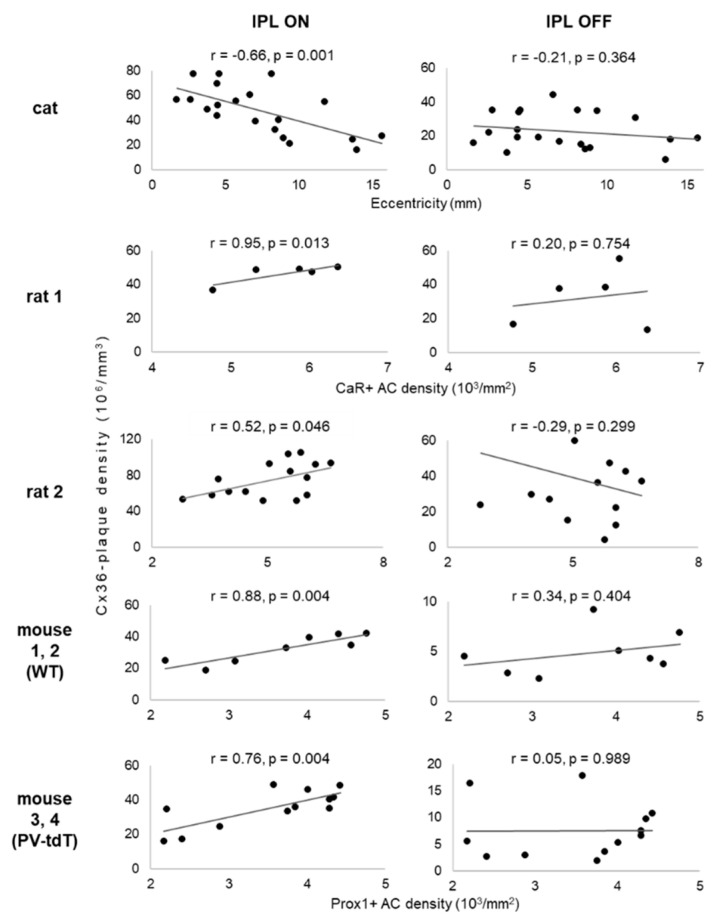
Relationship of volumetric connexin-36 plaque density to retinal position in the ON- and OFF-sublaminae of the inner plexiform layer of mammalian retinas. Each scatterplot shows data from several locations within one or more retinas treated the same way. Retinal location is indicated by the distance from the area centralis for cat retina (**top** row). For rodent retinas, retinal location is indicated by the areal densities of amacrine cells labelled by CaR or Prox1. Higher cell density indicates a higher sampling density of the retinal image; these regions must therefore be functionally more “central”.

Figure 9 compares the densities of Cx36 plaques in the ON and OFF sublaminae of the IPL at various retinal locations of cat, rat and mouse retinas. In the ON sublamina of cat retina, we found a strong, significant negative correlation with eccentricity (r = −0.66, *p* = 0.001), confirming our earlier observation [47]. However, plaque density in the OFF sublamina was uncorrelated to eccentricity (*p* = 0.364, Table 2). Interestingly, data from rodent retinas suggested the same relationship. In their ON sublaminae, Cx36 plaque density and the density of immunolabeled amacrine cells were positively correlated (*p* < 0.05, Table 2), implying that density decreased from central to peripheral regions. In contrast, no significant correlation was found in the OFF sublamina in either sample (*p* = 0.299, Table 2).

When data of the wild-type and PV-tdT mouse strains were pooled (Supplementary Figure S2), the same trends were evident (r = 0.77, *p* < 0.001 and r = 0.04, *p* = 0.856 for the ON and OFF sublaminae, respectively). This suggests that the Cx36 expression pattern was not affected by the Cre-mediated introduction of the tdT transgene. Another representation of the same data is when connexin density data are pooled by eccentricity category (centre, mid-centre, periphery, Supplementary Figure S1).

Finally, the independence of gap junctional connections in the OFF and ON sublaminae is further supported by the lack of significant correlation between the Cx36 plaque densities (*p* > 0.05, Table 2) of these sublaminae. In conclusion, our data suggest a fundamental difference in the organization of gap junctional connections between the ON and OFF sublaminae of the IPL.

## 4. Discussion

The main goal of the present study was to compare the regional variation in the density of punctate connexin-36 immunoreactive structures between the ON and OFF sublaminae of the inner plexiform layer in the retinae of multiple species. The central result is that regional variation in gap junction density follows different rules in the two sublaminae; whereas gap junctions become less frequent towards the periphery in the ON sublamina, there is no such correlation in the OFF sublamina. Since we calculated densities per unit volume of tissue, our results cannot be ascribed to systematic variation of the thickness of the laminae.

Two auxiliary results will also be discussed here. One is the regular occurrence of large Cx36 plaques on certain cell bodies of the amacrine cell layer. An additional result concerns the identity of Prox1 immunoreactive neurons in the retina, which we will discuss first.

### 4.1. Conservative Expression of Prox1 in Major Cell Types of the Inner Nuclear Layer

The Prox1 homeodomain transcription factor is expressed during development in various tissues including the brain and retina [75,76]. In adult animals, the retinal localization of Prox1 is best known for mice. Dyer et al. [51] found that Prox1 is expressed in horizontal cells and certain amacrine cells, that Pérez De Sevilla Müller et al. [52] later demonstrated to be AII amacrines. The same authors have shown using G0α immunohistochemistry that most of the large population of Prox1-positive bipolar cells are of the ON type. Similar immunohistochemical data from adult specimens of other species are not known to us except for those of Dyer et al. [51], also stating that Prox1 is localized to bipolar cells and parvalbumin-containing AII amacrine cells in rat retina. Although the density range of Prox1 immunoreactive amacrine cells matches the densities of AII cells identified by other methods in rats and in cats [57,77], further investigation is required to identify the population of Prox1 immunoreactive amacrines that do not express typical neurochemical markers of AII amacrine cells.

Our data provide evidence that the expression pattern of Prox1 is similar in carnivores and rodents as far as three major neuron types of the INL contain this protein. This corroborates the idea that the Prox1-dependent mechanism of retinal cell differentiation is conserved across mammalian species [51,78]. Moreover, the presence of Prox1 in both A- and B-type horizontal cells of cats [79] taken together with their neurochemical similarity [54,56,58,80,81] implies that despite their morphological differences, horizontal cell types differentiate late in development [82].

### 4.2. Somatic Gap Junctions of Retinal Amacrine Cells

Somato–somatic gap junctions are rather the exception than the rule in nervous tissue [83,84]. One notable example is the mesencephalic nucleus of the trigeminal nerve, where perikarya of primary afferent neurons form functional pairs or small clusters by way of electrical synapses [85,86,87].

In the neuropils of retina, gap junctions are most often localized on the dendritic or axonal processes of neurons [84,88] and they have also been discovered in the optic nerve head where they interconnect certain axons [89]. Somato–somatic gap junctions on AII amacrine cell bodies have been known for a while, although they have received little attention. They were first described in an electron microscopic study of the AII amacrine network of the cat retina [90], where large gap junctions were seen between AII cell bodies, sometimes connected by long appendages only a few hundred nanometres thin. It is reasonable to assume that the prominent, solitary Cx36 plaques seen on AII cell bodies in our material (Figure 6 and Figure 7) are likely the light-microscopic correlates of these gap junctions. The fact that we see them in multiple species taken together with earlier observations in rat [28,68], human [28,39] and feline [47] retinas suggest that they are an ubiquitous feature among mammals.

Somatic Cx36 plaques were almost exclusively associated with Prox1 immunoreactive amacrine cells, although in some cases, the neighbouring soma (and putative synaptic partner) was not labelled. One simple explanation is that the putative synaptic partner is from a different cell type, an example of which is shown in Figure 7. Another possibility is, without excluding the previous one, that the synaptic partner is located further away, attached through a process emanating from the cell bodies; such processes may be up to 10–15 μm long [90,91], which amounts to the width of multiple amacrine cell bodies and is in the order of the mean nearest neighbour distance of AII cells [77,92]. It is, however, unclear at present whether the processes seen in the electron microscope to connect AII somata through gap junctions [90] are identical to those thin, short dendrites that are seen at the light-microscopic level to attach directly to the cell bodies [91].

The frequency of occurrence of somatic gap junctions is another difference between the electron microscopic reconstructions of Vardi and Smith [90] and what is observed by immunofluorescence microscopy. We counted that the average ratio of somatic Cx36 plaques and Prox1 positive amacrine cells is around 8–10% with little variation between species. Vardi and Smith [90], on the other hand, reported that “wherever AII somas abutted, they formed large gap junctions” and even those further apart were sometimes linked (although see [93]). Both techniques revealed that the somatic gap junctions of AII cells are particularly large and therefore, the reason for the difference in occurrence is unlikely to be the lower resolution of the light microscope. There seem to be two possibilities worth pursuing. First, that a significant proportion of the somatic AII gap junctions failed to react with the anti-Cx36 antibody used. We are, however, not aware that any other connexin isoform has been observed to form plaques at this site so far. Alternatively, the number of somatic gap junctions or their detectability as plaques may vary with the functional state of the retina. Light adaptation [94] or injury [95,96] are known to affect functional coupling, which may be accompanied by detectable structural changes given that the half-life of connexin protein is in the order of a few hours [97,98].

AII amacrine cells are coupled extensively through Cx36-containing gap junctions [99,100] located on their arboreal dendrites in the ON sublamina of the IPL [101,102]. Somatic coupling could simply add another route to this connectivity without conferring an additional function. However, morphologically accurate modelling suggests that AII cells are electrotonically not compact and thus, somatic and dendritic inputs may be processed differently [103]. It is therefore worthwhile considering high-conductivity somatic gap junctions when modelling the biophysical properties of AII cells.

### 4.3. Different Scaling Principles of Connexin-36 Gap Junction Density in ON and OFF Circuits with Retinal Position

The present work builds on previous research on the distribution of electrical synapses in the cat retina [47]. Although cats and rodents have adapted to quite different lifestyles and, accordingly, their retinas and visual systems are differently specialised, the similarities revealed here suggest that we have encountered regularities generalizable for mammalian retinas.

The role of gap junctional connections in the retina is generally characterized by the formation of functional syncytia in which electrotonic membrane potential changes and potentially other signal carriers can propagate in the lateral direction. In this light, the density of gap junctions can be interpreted as indicating the strength of interconnections in these syncytia. As gap junctions are involved in several parallel processing pathways [22], multiple electrically coupled networks can coexist and be superimposed. The anatomical separation of the ON and OFF sublaminae of the INL allowed us to observe the large-scale differences between gap junctions contributing to the ON- and OFF-pathways. Our results confirmed that in mammalian species, the ON sublamina contains significantly more Cx36 gap junctions than the OFF sublamina [28]. It is widely believed that this difference is mainly due to the gap junctions formed by AII amacrine cells with ON-cone bipolar cells and among themselves [13,99,100,104]. Although the estimated number of gap junctions formed by each AII cell varies between a few dozen (estimates from electron microscopy [93,105,106]) and over a hundred (estimates using Cx36 immunofluorescence [47,104,107]), the fact that AII cells are the most frequent type of amacrine cell [108] makes it likely that they are the main contributors to the high density of Cx36 plaques detected in the ON sublamina. Furthermore, calculations based on comparison of AII amacrine cell and Cx36 plaque densities in the cat retina [47] have suggested that at least half of the Cx36 plaques in the ON-sublamina belong to other cell types [18,21,22,36,41,42,43,44,45,46].

The positive correlation of Cx36 plaque density with AII amacrine cell density may not come as a surprise because of the large contribution of this cell type to gap junctional connections. It is, however, worth considering that for most retinal cell types, lower cell density is compensated by a larger dendritic (or axonal) field diameter, so that the potential for synaptic connections is maintained. Why would then the density of synaptic contacts diminish with cell density? A plausible explanation may be gained from the observation that larger dendritic fields are typically sparser [109], a regularity that also applies for AII cells [91]. As a result, the meshwork of dendrites of a given type of neuron tends to be more loosely knit in the periphery. Importantly, it is also known for several retinal cell types that the number of synapses formed by a cell scales linearly with the available cell membrane area (and not with the area covered by the dendritic field) [110,111,112,113]. As a result, synapses of smaller (more central) cells are expected to be present at higher volumetric density in the neuropil than the synapses of larger (more peripheral) cells.

If the above line of reasoning is accepted, it is more difficult to explain why gap junction density does not decrease towards the periphery in the OFF-sublamina. An important contributing factor may be the diversity of the Cx36 expressing cell population. Indeed, gap junctions of the OFF-sublamina fall into four categories including those formed by ganglion cells with either RGC neighbours or nearby amacrine cells [34,35,36,37]—the population that connects amacrine cells to one another and a fourth population that exists between bipolar cells [38,39,40]. We can safely assume that at large, the density and arborization of the contributing cell types underlies the same scaling principles as outlined for the ON-sublamina. However, the dendritic fields of different cell types may well scale by different factors with eccentricity and the structure of the dendritic trees may also change differently with size [109,112]. Ultimately, the superposition of a variety of such systems would obscure eccentricity-dependent regularities that may be present in the connectivity of a single cell type.

An alternative hypothesis for the different scaling of Cx36 density in the OFF sublamina may be based on a recent study [114]. Here, the authors tested the presence of functional electrical synapses on ON- and OFF-bipolar cells by using in vitro electrophysiology and the gap junction blocker meclofenic acid in rat retinas. Surprisingly, the measurements suggest that the gap junctions of OFF-bipolar cells may not be functional electrical synapses. Instead, they could, for instance, serve metabolic functions or pass signal molecules between coupled cells. In any case, the distribution of non-synaptic gap junctions on the neurites may underlie different scaling rules compared to those that apply for synaptic contacts. If a sufficiently large proportion of Cx36 gap junctions in the OFF sublamina are of this kind, our measurements of the gross gap junction density may also be affected.

Finally, one might contemplate the idea that the regularities seen in the ON sublamina are generalized across species. This may suggest some general mechanism that scales gap junction density to cell density. To test this idea, we pooled the Cx36 and Prox1-positive amacrine cell density data of mice and rats. Supplementary Figure S2 shows that indeed, the correlation for IPL-ON observed within species is retained (r = 0.77, *p* < 0.0005), and there is no significant correlation in the OFF sublamina. It is, however, worth noting that cross-species differences still exist because in the region where AC densities of the two species overlapped, the Cx36 densities were lower on average in mouse than in rat samples.

### 4.4. Concluding Remarks

Although the ON and OFF responses of visual neurons are conceptually symmetrical, the pathways that implement them in the retina are fundamentally different (see e.g., [115] for review). There is abundant evidence that the visual response properties of ON and OFF neurones are also different in many ways [116,117,118]. Typically, these so-called ON/OFF asymmetries are investigated at a given, limited retinal location, and results obtained from central and peripheral parts of the retina could sometimes not be reconciled [116,119,120]. This points to the existence of region-specific ON/OFF asymmetries in the retina [121], which must eventually be rooted in regional differences of synaptic circuitry. Our study demonstrates large-scale differences between gap junctions involved in the ON and OFF networks of the retina potentially contributing to region-specific functional ON/OFF asymmetries.

## Figures and Tables

**Table 1:** List of key resources.

Reagent or Resource	Source	Identifier
**Antibodies**		
rabbit polyclonal anti-calretinin	Merck Hungary, Budapest, Hungary	AB 5054
mouse monoclonal anti-connexin-36	Merck Hungary, Budapest, Hungary	MAB 3045
rabbit polyclonal anti-Prox1	Merck Hungary, Budapest, Hungary	ABN 278
mouse monoclonal anti-parvalbumin	Swant, Burgdorf, Switzerland	PV 235
mouse monoclonal anti-calbindin	Synaptic Systems, Göttingen, German	214011
biotinylated anti-mouse IgG (H + L)	Vector Laboratories, Burlingame, CA, USA	BA 2001
streptavidin-Alexa Fluor 488 conjugate	Invitrogen, Waltham, MA, USA	S 32354
donkey anti-mouse IgG (H + L) Alexa Fluor 488 conjugate	Jackson ImmunoResearch, West Grove, PA, USA	715-545-151
donkey anti-rabbit IgG (H + L) Alexa Fluor 594 conjugate	Jackson ImmunoResearch, West Grove, PA, USA	711-585-152
goat anti-rabbit IgG (H + L) Texas Red conjugate	Jackson ImmunoResearch, West Grove, PA, USA	111-075-003
**Animals**		
domestic cat (*Felis catus*)	Animal house of the Institute of Physiology, Medical School, University of Pécs, Hungary	N/A
rat (*Rattus norvegicus*), Wistar strain	Animal house of the Institute of Physiology, Medical School, University of Pécs, Hungary	N/A
mouse (*Mus musculus*), C57BL/6J strain	The Jackson Laboratory, Bar Harbor, USA	000664
mouse (*Mus musculus*), PV-Cre line	The Jackson Laboratory, Bar Harbor, USA	B6.129P2-Pvalb^tm1(cre)Arbr^/J, JAX #017320
mouse (*Mus musculus*), tdTomato line	The Jackson Laboratory, Bar Harbor, USA	B6.Cg-Gt(ROSA)26Sor^tm9(CAG-tdTomato)Hze^/J, JAX #007909
**Equipment**		
Zeiss LSM 710 confocal laser scanning microscope	Carl Zeiss, Jena, Germany	
**Software**		
Fiji	https://imagej.net/software/fiji/, accessed on 27 July 2018	N/A
Imaris 8.12	Oxford Instruments, Zurich, Switzerland	N/A
SPSS 24.0	IBM Corporation, Armonk, NY, USA	N/A
Excel for Office 365	Microsoft, Redmond, WA, USA	N/A

**Table 2:** Relationship of connexin-36 plaque density and retinal position in mammalian retinas.

Ample (Number of Retinal Locations)			Cat (*n* = 20)	Rat 1 (*n* = 5)	Rat 2 (*n* = 15)	Mouse 1 and 2, Wild Type (*n* = 8)	Mouse 3 and 4, PV-tdT (*n* = 12)
Marker used for measurement of cell density			none	CaR	Prox1	Prox1	Prox1
Measure of retinal position			Eccentricity (mm)	Amacrine cell density (mm^−2^)			
Mean ± SD			7.34 ± 4.01	5766 ± 598	5085 ± 1139	3687 ± 904	3520 ± 836
IPL sublamina	OFF	Cx36-density (1/mm^–3^)	22.8 × 10^6^ ±10.5 × 10^6^	32.3 × 10^6^ ±17.4 × 10^6^	38.7 × 10^6^ ±24.5 × 10^6^	4.84 × 10^6^ ±2.29 × 10^6^	7.55 × 10^6^ ±5.53 × 10^6^
		Correlation with retinal position	r = −0.21 *p* = 0.364	r = 0.20 *p* = 0.754	r = −0.29 *p* = 0.299	r = 0.34 *p* = 0.404	r = 0.05 *p* = 0.989
	ON	Cx36-density (mm^–3^)	47.7 × 10^6^ ±11.8 × 10^6^	46.2 × 10^6^ ±5.55 × 10^6^	74.3 × 10^6^ ±19.4 × 10^6^	32.3 × 10^6^ ±13.2 × 10^6^	35.0 × 10^6^ ±15.5 × 10^6^
		Correlation with retinal position	r = −0.66 *p* = 0.001	r = 0.95 *p* = 0.013	r = 0.52 *p* = 0.046	r = 0.88 *p* = 0.004	r = 0.76 *p* = 0.004
Difference of Cx36-density between IPL OFF and IPL ON			t = −10.05 *p* < 0.001	t = −2.44 *p* = 0.024	t = −5.62 *p* < 0.001	t = −47.95 *p* < 0.001	t = −24.33 *p* < 0.001
Correlation of Cx36-density between IPL OFF and IPL ON			r = 0.11 *p* = 0.325	r = 0.33 *p* = 0.587	r = 0.42 *p* = 0.121	r = 0.54 *p* = 0.463	r = 0.77 *p* = 0.075

## Data Availability

Raw data that are not reported in the paper are available on request from the corresponding author, P.B.

## Supplementary Materials

The following are available online at https://www.mdpi.com/article/10.3390/cells10092396/s1, Figure S1: Data of Prox1 and Cx36 immunolabelled mouse retinas from Figure 9 plotted with eccentricity categories (centre, mid-centre, periphery) on the abscissa, Figure S2: Data of Prox1 and Cx36 immunolabelled rat and mouse retinas from Figure 9 plotted together for comparison.
