# Supplementary material for: Regional Variation of Gap Junctional Connections in the Mammalian Inner Retina
Source: Cells. 2021 Sep 12;10(9):2396. doi: 10.3390/cells10092396 (PMC8466939; doi:10.3390/cells10092396)
Supplement: Supplementary file 1 [file cells-10-02396-s001.zip › cells-1351548-SI.pdf]

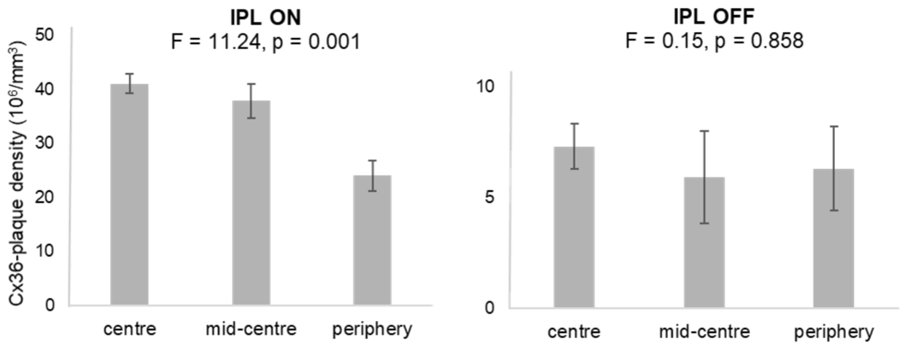

Supplementary Figure S1. *Data of Prox1 and Cx36 immunolabelled mouse retinas from Figure 9 plotted with eccentricity categories (centre, mid-centre, periphery) on the abscissa. ANOVA shows significant differences between locations in the ON sublamina but not in the OFF sublamina of the IPL. In the ON sublamina, there appears to be a constant decrease of connexin density from centre to periphery, which is less steep between centre and mid-centre. This can be explained by the fact that samples included all four retinal quadrants and the change in overall cell density is steeper along the dorso-ventral axis than along the naso-temporal axis.*

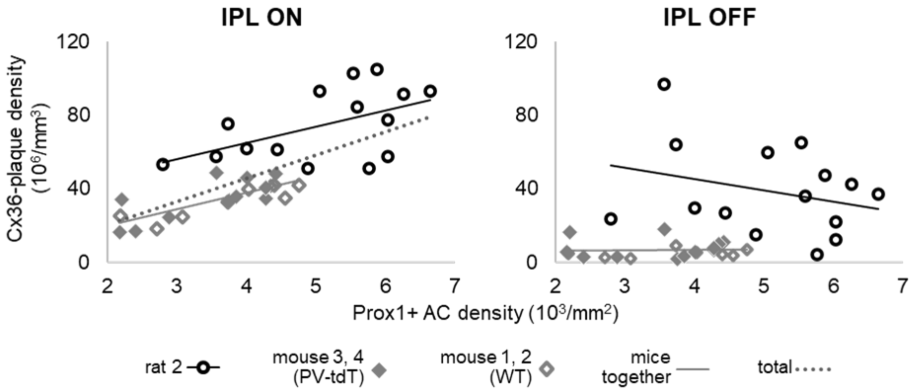

Supplementary Figure S2. *Data of Prox1 and Cx36 immunolabelled rat and mouse retinas from Figure 9 plotted together for comparison. The graphs show the relationship of volumetric connexin-36 plaque density to retinal position in the ON- and OFF-sublaminae of the inner plexiform layer, where higher amacrine cell density indicates more central location. The data of the wild-type and PV-tdT mouse strains (grey markers) overlap completely. The connexin and AC densities of the rat sample (black markers) are both higher on average. Pooled data of the two mouse strains ("mice together"), as well as pooled data of rat and mouse samples ("total") show the same trends as those of individual data sets, i.e. significant correlation in the ON sublamina and no significant correlation in the OFF sublamina of the IPL. Correlation coefficients for the ON sublamina are as follows. For "mice together", r = 0.77, p <0.001; for "total" , r = 0.77, p <0.0005. Correlation coefficients for the ON sublamina are as follows. For "mice together", r = 0.04, p = 0.856; for "total" , r = 0.32, p <0.062.*
